# Exploring the Binding Affinity of the ARR2 GARP DNA Binding Domain via Comparative Methods

**DOI:** 10.3390/genes14081638

**Published:** 2023-08-17

**Authors:** Janine Rieger, Michael Fitz, Stefan Markus Fischer, Niklas Wallmeroth, Hector Flores-Romero, Nina Monika Fischer, Luise Helene Brand, Ana J. García-Sáez, Kenneth Wayne Berendzen, Virtudes Mira-Rodado

**Affiliations:** 1 Center for Plant Molecular Biology (ZMBP), Tübingen University, 72076 Tübingen, Germany; 2 Interfaculty Institute of Biochemistry (IFIB), Tübingen University, 72076 Tübingen, Germany; 3 CECAD Research Center, Institute of Genetics, Cologne University, 51069 Cologne, Germany; 4 Institute for Bioinformatics and Medical Informatics, Tübingen University, 72076 Tübingen, Germany

**Keywords:** ARR2, GARP, transcription factor, DPI-ELISA, FCS, MST, structural modeling, reporter gene

## Abstract

Plants have evolved signaling mechanisms such as the multi-step phosphorelay (MSP) to respond to different internal and external stimuli. MSP responses often result in gene transcription regulation that is modulated through transcription factors such as B-type Arabidopsis response regulator (ARR) proteins. Among these proteins, ARR2 is a key component that is expressed ubiquitously and is involved in many aspects of plant development. Although it has been noted that B-type ARRs bind to their cognate genes through a DNA-binding domain termed the GARP domain, little is known about the structure and function of this type of DNA-binding domain; thus, how ARRs bind to DNA at a structural level is still poorly understood. In order to understand how the MSP functions in planta, it is crucial to unravel both the kinetics as well as the structural identity of the components involved in such interactions. For this reason, this work focusses on resolving how the GARP domain of ARR2 (GARP2) binds to the promoter region of *ARR5*, one of its native target genes in cytokinin signaling. We have established that GARP2 specifically binds to the *ARR5* promoter with three different bi-molecular interaction systems—qDPI-ELISA, FCS, and MST—and we also determined the K_D_ of this interaction. In addition, structural modeling of the GARP2 domain confirms that GARP2 entails a HTH motif, and that protein–DNA interaction most likely occurs via the α_3_-helix and the N-terminal arm of this domain since mutations in this region hinder ARR2’s ability to activate transcription.

## 1. Introduction

Information processing, metabolism, self-maintenance, and self-replication signaling networks proceed through molecular interactions. These networks occur through a series of binding, recognition, and dissociation events between biological molecules formed primarily by non-covalent complexes [1,2]. The strength of such bi-molecular interactions is determined by the binding affinity of the two molecules and is encompassed in physio-chemical terms such as the dissociation constant, K_D_ [3]. The K_D_ is a constant that quantifies the binding affinity of the interaction of two (or more) molecules that bind reversibly. It is represented by the concentration of the free protein that occupies half of the overall sites of the second protein at equilibrium [3]. Both direct and indirect methods have been developed to determine K_D_s; some examples are NMR spectroscopy, ultrafiltration and electrophoretic methods, DNA–protein interaction-enzyme-linked immunosorbent assay (DPI-ELISA), differential scanning calorimetry, isothermal titration calorimetry (ITC), fluorescence correlation spectroscopy (FCS), surface plasmon resonance (SPR), and microscale thermophoresis (MST) [1,4,5,6]. Moreover, more traditional methods such as electromobility shift assays (EMSA) have been used to estimate dissociation constants [7].

DNA–protein interaction-enzyme-linked immunosorbent assay is a powerful method that possesses several advantages compared to other in vitro methods for the analysis of protein–DNA interactions [8,9]. ELISA is a method to determine bi-molecular interactions and was first published by two Swedish groups in 1971; it was based on an enzyme-mediated color reaction used to detect the interaction between antigens and antibody enzyme conjugates [10,11]. This was then modified to probe DNA–protein interactions where DNA is usually bound to the microtiter plate [12]. Further use of fluorophore-protein fusions allowed for the direct fluorometric detection of the protein–DNA complex (qDPI-ELISA), shortening the protocol and increasing the linear range of detection [13].

Fluorescence correlation spectroscopy is a technique that monitors how fluorescently labeled molecules diffuse through a small observation volume by measuring their fluorescence emission over time and analyzing the autocorrelation of the signal [14]. As molecule’s diffusion is usually linked to their molecular mass, this technique has been widely used to study bi-molecular interactions, where free and thus smaller molecules move faster through the observation volume than molecules that are bigger due to complex formation [15,16,17,18]. In the past years, FCS has slowly been introduced in the plant field; Li and colleagues [15] reviewed very nicely how FCS has proven to be a powerful tool by which to understand the organization of plant cell membranes and the dynamics and interactions of membrane proteins. Outside the plant membrane, FCS has emerged as an influential tool by which to study bi-molecular interactions concerning protein oligomerization, protein–protein, or protein–DNA and –RNA interactions [19,20,21,22,23,24,25].

Microscale thermophoresis is a relatively young technique that monitors the motion of molecules in a temperature gradient. It is based on the Ludwig–Soret effect, also called thermophoresis, that describes the directed movement of molecules induced by a temperature gradient in an aqueous solution [26]. The thermophoretic mobility of a molecule is very sensitive to the molecule–solvent interface, which is embedded in the size and charge of the molecule, and which, of course, can vary with its own conformational state, but will also change when associated with other molecules [26]. Thus, MST provides an indirect method for monitoring bi-molecular interactions by tracking the movement of a fluorescently labeled molecule before, during, and after a temperature gradient is created with an infrared laser. Ultimately, MST technology enables the calculation of binding affinity constants such as the dissociation constant by analyzing how the thermophoretic mobility of the fluorescently labeled molecule is affected by different concentrations of an unlabeled interacting partner [5,27].

In addition to the methods described above, a better understanding of how bi-molecular interactions occur can be achieved via structural-based computational approaches that have been developed to model complexes and determine binding free energies. Computational modeling is inexpensive and can be repeated often without any additional costs (when compared to experimental approaches) to determine three-dimensional structures such as X-ray diffraction or nuclear magnetic resonance (NMR) analysis. Furthermore, with molecular dynamics, one can add flexibility to all-atom models and can thus try to add a more realistic protein structure surrounded by water molecules and ions. However, setting up molecular dynamic simulation protocols requires a lot of fine-tuning and depends on the quality of pre-existing protein or DNA structures via NMR or X-ray analysis for them to be reliable [28]. Thus, once provided with high-quality preceding structures, one can use structural modeling to obtain a quite-reliable idea of how a certain protein may actually fold, allowing predictions on putative contact residues between molecules [29].

Plants have evolved signaling mechanisms such as the Multi-Step Phosphorelay to respond to different internal and external stimuli. The functionality of signaling mechanisms such as the MSP are strongly dependent on bi-molecular interactions. In Arabidopsis, the MSP is composed of three types of proteins: Arabidopsis histidine kinases (AHKs); phosphotransfer proteins (AHPs); and response regulators (ARRs). Here, signal perception is carried out by the AHKs, which initialize a phosphorylation relay that ends up with the activation of the ARRs [30,31]. In Arabidopsis, ARRs are subdivided into three types (A-, B-, and C-type ARRs) depending on their function and protein structure. While all ARRs have a conserved receiver domain containing the aspartate residue responsible for phosphorylation, the output domain among the different ARR subgroups differs substantially [32]. In A- and C-type ARRs, this domain is quite short, whereas in the case of B-type ARRs (ARR-Bs), the output domain is more complex and more typical of transcription factors since it contains structures such as nuclear localization signals (NLS), a transactivation domain (TA), and a DNA-binding domain. This DNA-binding domain has also been called the B-motif [33] or the GARP domain [34,35] since it is part of the GARP family of Myb-like transcription factors (TFs) named after different TFs containing this motif: GOLDEN2 from maize; ARRs from Arabidopsis; and PSR1 from *Chlamydomonas reinhardtii* [34,35]. The GARP domain is also present in other Arabidopsis proteins such as the PHR1 [36], KANADI [37], and pseudo response regulators [32]. The GARP domain family of TFs is subdivided into two major groups: group I includes the GOLDEN2, KANADI and B-type RRs; group II includes PHR1 and PSR1 [34,35].

The GARP domain of B-type ARRs resembles both Myb repeats and homeodomains, and it represents a fundamental DNA-recognition unit observed in many DNA binding proteins present in all eukaryotes [33,38,39,40,41,42]. The Myb DNA binding domain generally consists of up to four helix–turn–helix (HTH) repeats responsible for DNA recognition and transactivation [41,43]. Similarly to the Myb motif, the homeodomain also contains a HTH motif as a DNA binding domain (DBD) [40]. The engrailed (ENG) protein of Drosophila is a prototypic example of homeodomain protein [38]. Because the GARP domain of B-type ARRs resembles a single repeat of the classic Myb domain, it is thus considered to be a plant single Myb-related motif. The Myb-like and the GARP family all bind short sequences revolving around a 5′-*GAT*/*ATC*-3′ consensus sequence. Group II GARP proteins bind to a gapped form of this motif 5′-*GnAT*/*ATnC*-3′ [44], while group I GARP proteins such as the B-type ARRs bind to another version of this sequence, 5′-*RGATY*-3′, commonly termed the cytokinin response motif (CRM) [33,45,46,47,48,49,50].

Due to their structural similarity, it is generally accepted that all B-type ARRs function as TFs, although direct roles as transcriptional activators has only been demonstrated for ARR1, ARR2, ARR10, ARR11, and ARR18 [45,47,48,51,52,53,54,55]. Of the eleven B-types ARRs in *A*. *thaliana*, five members have been shown to mediate cytokinin signaling responses such as root elongation, namely, ARR1, ARR2, ARR10, ARR11, and ARR12 [52]. *ARR2* is known to be expressed ubiquitously in *A*. *thaliana*, albeit to different degrees [56,57,58] and not surprisingly, is involved in many aspects of plant development and regulation [56,59,60,61,62,63]. In addition, ARR2 shows both positive and negative synergy with ARR1, ARR10, and ARR12 in root cytokinin signal transduction [52].

Despite the fact that plant Myb-related domains such as the B-type RR’s GARP motif appear in different plant transcription factors, little is known about their structure and how they function. For example, although the consensus target DNA sequence of many plant B-type RRs has been determined with various methods, to date, the mechanisms by which ARRs bind to DNA at a structural level have only been studied for the isolated GARP domain of ARR10 (GARP10) [33].

Based on our previous work, we are interested in ARR2 as it represents a close yet phylogenetically separated RR to ARR10, forming a similar but functionally divergent complementation group [52,62]. ARRs–DNA interaction studies have overlooked natural promoter targets. For this reason, our paper focusses on resolving how the GARP domain of ARR2 (GARP2) binds to the promoter region of *ARR5* (*ARR5p*), one of its native target genes. *ARR5* is an early cytokinin response gene that is directly activated by B-type ARRs, including ARR2 [55,56] via its common core target sequence 5′-*RGATY*-3′ [45,57].

We tested the efficiency of GARP2 to bind to a fragment of the *ARR5* promoter under three different bi-molecular interaction systems: qDPI-ELISA; FCS; and MST. By exploring multiple methods for the determination of a particular phenomenon, we can compensate for unknown, hidden variables that can influence the outcome. The three bi-molecular interaction approaches unequivocally demonstrated a specific binding affinity of the GARP2 to the *ARR5* promoter fragment, and we were able to determine the K_D_ for the interaction via MST. In addition, we present a structural 3D model of the GARP2 protein in complex with DNA. Structural modeling and transactivation assays determined that protein–DNA interaction most likely occur via specific amino acids in the α_3_-helix and the N-terminal arm of the GARP2 domain.

## 2. Results

### 2.1. Generation of GARP2 Fusion Proteins and Its DNA Target Sequences within the ARR5 Promoter

**Figure 1:**
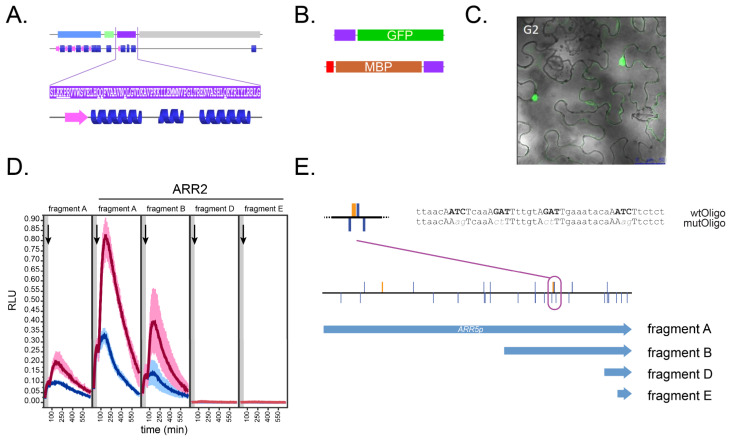
Schematic of the GARP2 domain and DNA target sites used in this work. (**A**) Scheme of the ARR2 protein and its three major domains: receiver domain (light blue); acidic domain (lime); GARP domain (purple); and P/Q domain (grey). The JPred secondary structural prediction for the entire ARR2 protein is shown with an enlarged view of the GARP2 domain showing a β-sheet (pink) and three α-helices (blue). Putative GARP2 NLS is underlined. (**B**) Scheme of the GARP2–eGFP and His–MBP–GARP2 fusion proteins generated in this work: GARP2 (purple); 8xHis Tag (red); eGFP (green); and MBP (brown) (**C**) Transient expression of GARP2–eGFP (G2) in tobacco leaves as descri bed in Veerabagu et al. [53] (**D**) Transactivation assay of *ARR5p::LUCm*^3^ promoter fragments (see **E**) with ARR2 as effector after cytokinin (red) or mock (blue) treatments. The grey bar represents the flash measurement prior to cytokinin addition. The black arrow marks the time point of hormone application. Light emission is given as relative LUC activity (RLU). The lighter area around the curves represents the standard error calculated from four technical replicates. (**E**) Schematic of the *ARR5* promoter region (5′→3′) containing putative B-type ARR CRM recognition sites (A/G)GAT(T/C) (blue bars) and *ECRM* motifs AAGAT(T/C)TT (orange bars); top bars have sense and bottom bars have antisense orientation. The region chosen for in vitro analyses is circled and its sequence given to the right: wild-type sequence (wtOligo) and the mutated sequence (mutOligo). The various *ARR5* promoter fragments used in D are shown as blue arrows.

In order to study the ability of the ARR2 GARP domain (Figure 1A) to interact with DNA, it was classically cloned into bacterial expression vectors to create two different GARP2 fusion proteins. First, GARP2 fused to the green fluorescent protein (GARP2–eGFP) for performing fluorescent-based DPI-ELISA assays; second, GARP2 fused to both an 8x-histidine peptide and a maltose binding protein tag (His–MBP–GARP2) for fluorescence correlation spectroscopy and microscale thermophoresis assays (Figure 1B).

We transfected tobacco leaves with the GARP2–eGFP fusion protein to determine its subcellular location (Figure 1C). ARR1 and ARR2 have been shown to contain nuclear localization signals near their GARP domains [45,57]. In addition, ARR10 also contains a functional NLS inside its GARP domain [33]. As can been seen in Figure 1C, GARP2 also localized primarily to the nucleus; thus, like ARR10, it must possess a functional NLS within it. The putative GARP2 NLS is underlined in Figure 1A.

In order to choose a DNA target motive for the GARP2–DNA interaction, we decided to investigate *ARR5*, a natural ARR2 target gene [46,55] that contains potential cytokinin response motif binding sites in its promoter region. As seen in Figure 1E, the *ARR5* promoter contains multiple copies of the CRM site 5′-(A/G)GAT(T/C)-3′ [46]. Beyond these core motifs, the *ARR5* also contains the extended cytokinin response motif (ECRM), which is an extended version of the CRM motif that has been shown to be required by some B-type ARRs such as ARR1 to mediate full transcriptional activation [64]. In order to determine which regions in the *ARR5p* are relevant for ARR2 binding in vivo, we created different *ARR5p* truncations (A, B, D, and E) and tested them for ARR2 activation in vivo (Figure 1D). Here, in planta transactivation assays in protoplasts were carried out as described by Wallmeroth and colleagues [55] using the different *ARR5p* fragments fused to firefly luciferase (*ARR5p::LUCm*^3^) as reporter genes and ARR2 as effector. Fragment A corresponds to the *ARR5* full-length promoter, beginning at −2254 bp with respect to the translation start codon. As shown in previous publications, our data confirms that the full-length *ARR5p* is cytokinin inducible and is hyper-activated when ARR2 is used as an effector. In comparison, fragment B (−1091 bp), although to a lesser extent, still elicits an ARR2-cytokinin dependent response. In conclusion, CRMs that are farther upstream of fragment B are apparently required for a full activation of *ARR5p*; nevertheless, fragment B contains a probable ARR2 target region in vivo. In contrast, fragments D (−198 bp) and E (−144 bp) had nearly no transcriptional activity. From these data, we opted for a region of the *ARR5* promoter to be used as target DNA for GARP2–DNA interaction studies. As seen in Figure 1E, this DNA section contains three CRMs and one ECRM and is therefore likely a good target of B-type ARRs. Thus, we chose the wild-type sequence as the native condition (termed wtOligo) and generated a mutated version wherein the highly conserved nucleotides *TC* of the CRM motive are replaced with *AG* (termed mutOligo) (Figure 1C). These wtOligo and mutOligo oligos were synthesized for bi-molecular interaction assays to test for binding by GARP2.

### 2.2. GARP2 Interaction with DNA Analyzed via qDPI-ELISA

**Figure 2:**
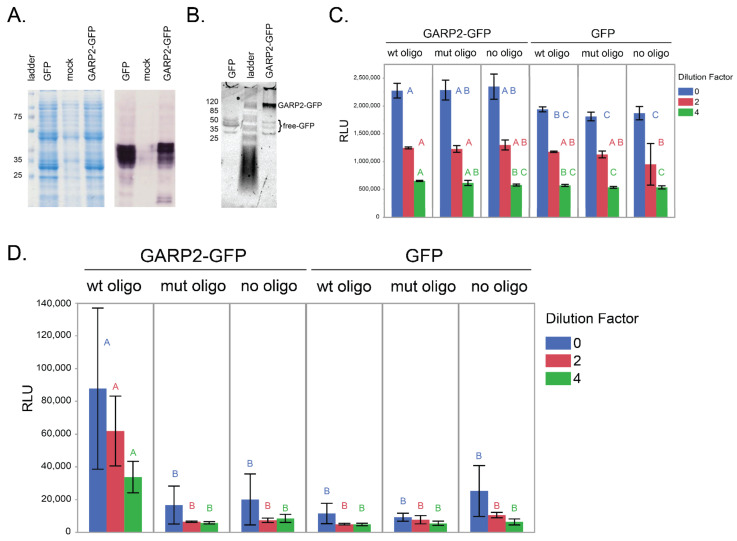
GARP2–GFP binds to DNA in a sequence-specific manner, as shown by qDPI-ELISA. (**A**) SDS-PAGE gel stained with Coomassie Brilliant-blue (**left**) and immunoblot using a GFP-specific antibody (**right**) of crude extracts from bacteria expressing GARP2–eGFP and eGFP proteins after IPTG induction. mock: control, i.e., non-transformed bacteria. (**B**) Native gel of GFP and GARP2–GFP illuminated with UV light to detect GFP fluorescence. (**C**) GFP emission of GARP2–GFP and GFP loaded to the 384-well plate before washing. (**D**) GFP emission of GARP2–GFP and GFP loaded to the 384-well plate after the first wash. no oligo = without oligo. Error bars depict the standard deviation of three technical replicates. Dilution factors: 0 (non-diluted), 2, and 4. After performing one-way ANOVA, significance classes were determined using Fischer’s least significant difference test, α = 0.01. Groups not connected by the same letter are significantly different.

In studying bi-molecular interactions, the DPI-ELISA method has the advantage of not requiring pre-enrichment or purification steps and can be chromogenic or fluorescent based [13]. To determine whether the GARP2 domain is sufficient to bind to specific regulatory elements of the *ARR5* promoter, we chose a fluorescent based DPI-ELISA strategy using GARP2 N-terminally cloned to eGFP (GARP2–eGFP). Both GARP2–eGFP and eGFP proteins were expressed in bacteria and isolated as cleared, crude extracts. Although no clearly visible bands could be observed in the Coomassie-stained gel after induction with IPTG, their presence in the extracts was confirmed via immunoblot analysis and both fusion proteins migrated as expected under denaturing conditions (Figure 2A). It is important to note that even if the proteins are only expressed to moderate amounts, based on previous work, proteins at very low concentrations should be analyzable with DPI-ELISA [12,13]. Under non-denaturing conditions, we could observe that the GARP2–GFP sample also contained free GFP that was present only in low quantities compared to the GARP2–GFP fusion protein (Figure 2B).

For the qDPI-ELISA assay, biotinylated dsDNA oligos (wtOligo and mutOligo) were immobilized to a streptavidin-coated ELISA plate. After protein extraction and quantification of the GFP amount by measuring fluorescence, GARP2–eGFP and eGFP extracts were loaded onto the plate in three dilution factors (Figure 2C,D). Figure 2C shows GFP emission levels to demonstrate that, both the GARP2–eGFP protein and the eGFP control were equally loaded before washing. After one wash, we consistently observed that only the GARP2–GFP with wtOligo was retained in the wells, proportional to the dilution scheme. All other combinations led to levels near or at the no-oligo background level. The specificity of the binding was confirmed via the mutOligo containing mutations in the CRM and ECRM motifs. This indicated that the GARP2 domain is fully functional and capable of binding specific CRM recognition sites within the *ARR5* promoter.

### 2.3. GARP2 Interaction with DNA Determined by Fluorescence Correlation Spectroscopy

**Figure 3:**
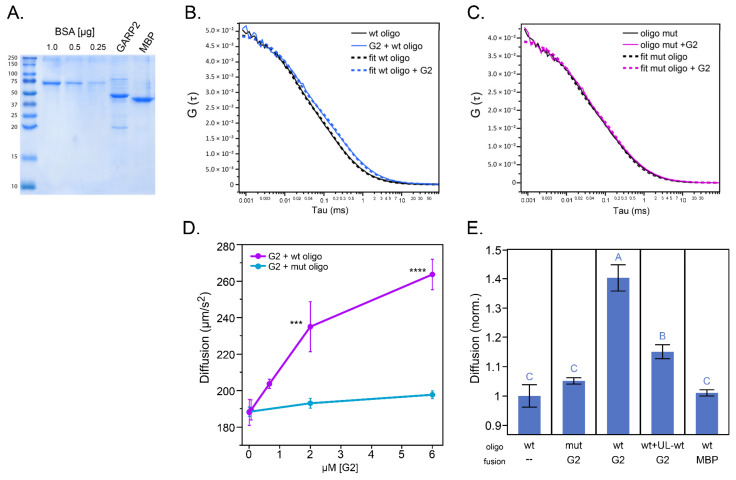
His–MBP–GARP2 binds to DNA in a sequence-specific manner as shown via fluorescence correlation spectroscopy assays. (**A**) Representative Coomassie-stained protein gel of purified His–MBP–GARP2 and His–MBP compared to BSA standards. His–MBP–GARP2 and His–MBP bands that are expected to be around 57 and 40 kDa, respectively. (**B**) Autocorrelation curves of the alexa647-labeled wtOligo (50 nM) in the presence or absence of purified unlabeled His–MBP–GARP2 protein (G2; 6 µM). (**C**) Autocorrelation curves of the alexa647-labeled mutOligo (50 nM) in the presence or absence of purified unlabeled His–MBP–GARP2 protein (G2; 6 µM). (**D**) Diffusion coefficient of target labeled oligos in the presence of different concentrations of purified unlabeled His–MBP–GARP2 protein (G2) (0, 0.066, 0.66, 2, and 6 µM). Significance was determined via one-way ANOVA, α = 0.05. *** ≥ 0.01; **** ≥ 0.001. At 2 µM G2 concentration, *p* ≥ 0.0065 and at 6 µM, *p* ≥ 0.0002. (**E**) Normalized (wtOligo alone as the value 1) diffusion values for wtOligo (wt) and mutOligo (mut) upon addition of His–MBP–GARP2 (G2) protein (6 µM), un-labeled wtOigo (UL-wt), or His–MBP (MBP; 6 µM). Alexa 647-labeled oligos were used at 50 nM and unlabeled wtOligo was 500 nM. Technical replicates *n* = 3; +/− standard deviation. Significance classes were determined via the Tukey–Kramer HSD test, α = 0.05; groups not connected by the same letter are significantly different. (**E**) Normalized (wtOligo alone as the value 1) diffusion values for wtOligo (wt) and mutOligo (mut) upon addition of His-MBP-GARP2 (G2) protein (6 µM), un-labeled wtOigo (UL-wt) or His-MBP (MBP; 6 µM). Alexa 647-labelled oligos were used at 50 nM and unlabeled wtOligo was 500 nM. Technical replicates *n* = 3; +/− standard deviation.

In FCS, we analyze the diffusion time required by fluorescently labeled molecules to pass a small observation volume [14]. As diffusion time in solution is mainly dependent on the mass of the molecules, in order to enhance the impact of GARP2 domain interaction on DNA/Oligo diffusion, we increased the GARP2 molecular weight by adding both a polyhistidine- and an MBP-tag (His–MBP–GARP2). Both His–MBP–GARP2 and the control protein, His–MBP, were expressed in bacteria, purified using Ni–NTA beads, and quantified via direct comparison to BSA standards (Figure 3A).

To obtain quantitative information on the ability of GARP2 to form stable complexes with DNA, we compared the autocorrelation curves of alexa647-labeled oligonucleotides (wtOligo and mutOligo) in the presence and absence of His–MBP–GARP2 non-labeled protein (Figure 3B,C). The observed shift in the decay of the wtOligo’s autocorrelation curve (Figure 3B) is correlated to its binding to the GARP2 protein, which results in a reduction of the oligo’s mobility. In addition, this interaction seems specific to the wtOligo since the diffusion time of the mutOligo was not affected by the addition of the GARP2 protein (Figure 3C). Thus, consistent with the DPI-ELISA assay, we were able to determine via FCS that GARP2 binds to the wtOligo and not the mutOligo.

In order to further characterize this interaction, we measured the diffusion time of the labeled oligos at different His–MBP–GARP2 protein concentrations (Figure 3D). In agreement with our previous data, the diffusion coefficient of the wtOligo, but not the one of the mutOligo, is significantly increased upon incubation with His–MBP–GARP2 protein in a concentration dependent manner. To test the reversibility of this interaction, we incubated the samples with an excess of unlabeled wtOligo in a competition experiment (Figure 3E). As expected, the diffusion coefficient of the labeled wtOligo in the presence of His–MBP–GARP2 was reduced upon the addition of the non-labeled one, indicating that the interaction is reversible. Finally, we analyzed the impact of the His–MBP protein on the wtOligo’s diffusion, where no significant changes were observable, suggesting that the interaction of the protein to the wtOligo occurs only when the GARP2 domain is present. Altogether, FCS analysis corroborates that the GARP2 domain binds to specific sites of the ARR5 promoter.

### 2.4. Quantification of the GARP2–DNA Binding Affinity via Microscale Thermophoresis

MST monitors the movement of a fluorescently labeled molecule through a temperature gradient (thermophoresis). If the fluorescently labeled molecule binds to a certain ligand, the speed at which it moves in the temperature gradient will change. MST can be used to monitor bi-molecular interactions and to compute the K_D_ of such interactions by measuring the thermophoretic movement of a particular fluorescently labeled molecule titrated against an unlabeled ligand [65,66].

For the MST analysis, we used again the 8xHis–MBP–GARP2 (GARP2) and 8xHis–MBP (MBP) fusion proteins but this time, we labeled the His tag with RED-tris-NTA dye [67]. Here, we monitored the migration of GARP2 and MBP fluorescently labeled molecules challenged against different concentrations of wtOligo and mutOligo. Fluorescence changes are examined in a specific illuminated volume where a temperature gradient is created. Fluorescence is constantly monitored as a function of time before, during, and after the IR-laser heat-gradient formation, generating what is called a thermophoretic time trace.

Due to the nature of the method, there are actually several different thermophoretic effects that can be observed from the same time traces. These are as follows: steady state levels before heat-gradient induction; a change in fluorescence during the heat-gradient formation called temperature-jump (T-jump) that is analogous to an acceleration in temperature change; thermophoresis (TmF) as a steady-state heat-gradient; inverse-temperature-jump (Inv. T-jump) when the IR laser is switched off; and back diffusion (BdF) as the molecules wander back into the illuminated volume. The ratio of change from one state to another comprises a thermophoretic metric, and each one carries information about the size charge, shape, and solvency of the molecules being studied and therefore information about the affinity and mechanism of binding. As it is impossible to know which thermophoretic metric is of most interest a priori [5,6,26,27,68,69]; moreover, it is likely undesirable to just choose one based on previous work. We decided to systematically test the different thermophoretic metrics (MST zones) under all experimental combinations.

All our experiments consisted of a 5 s initial fluorescence measurement, followed by 30 s of IR laser illumination for TJ and TmF. This was followed by five further seconds with the laser switched off for the back diffusion ITJ and BdF.

#### 2.4.1. Establishing MST Limits in the Different MST Zones

Thermophoresis values depend on the ratio of two observation points: the mean steady-state at an earlier time point, often called the “cold-fluorescence” or Reference (F_Reference_), and the mean steady-state at a later time point, called the “hot fluorescence” or Subject (F_Subject_) [5,26]. Thermophoresis is measured via the normalized fluorescence (F_n_), which can be defined as F_n_ = (F_Subject_/F_Reference_) × 1000 [5,27]. Although the zones mentioned above can be identified easily by the eye, their placement is of course guided by years of study that serve as base recommendations for their location [5,6,26,27,68].

**Figure 4:**
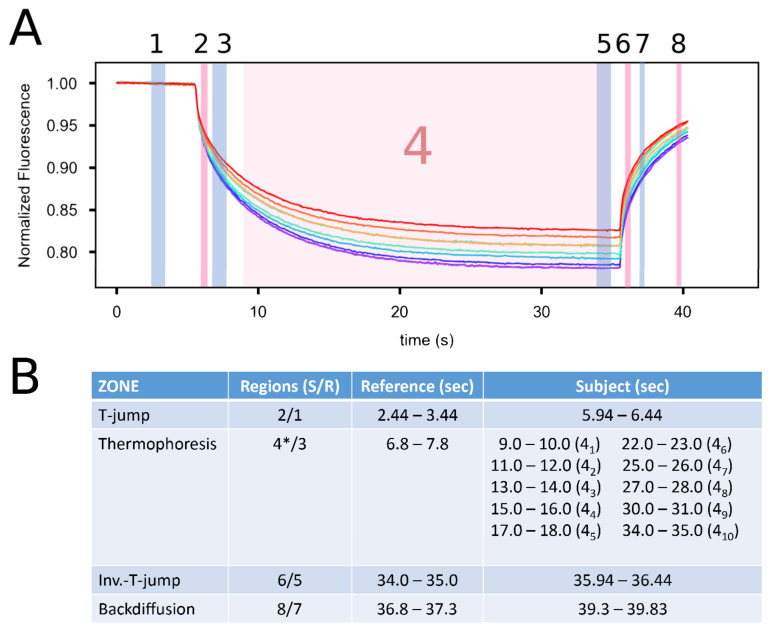
The delimitation of the four MST analysis zones used in this work. MST traces where F_Subject_ (hot fluorescence) and F_Reference_ (cold fluorescence) shown as pink and blue shaded zones, respectively according to Scheuermann 2016 [5] with time points are marked for the four different MST zones. The T-jump region comprises Subject 2 to Reference 1 (Region 2/1). The Thermophoresis, Subject 4 (light pink zone) to Reference 3. * Subject 4 includes multiple intervals (4_1_, 4_2_, 4_3_,…, 4_10_) that start at second 9 and end at second 35, i.e., region 4_1_/3 starts at Reference 3 and ends at Subject 4_1_ (second 9 to 10); similarly, region 3–4_10_, comprises Reference 3 to Subject 4_10_ (second 34 to 35). The inverse T-jump (Inv. T-jump) is Subject 6 to Reference 5. The back diffusion region examines the regions Subject 8 to Reference 7. (**A**) Exemplary representation of fluorescence trace profiles over time shown in rainbow colors with the different Reference and Subject time points. (**B**) Region limits for the four MST zones and their definitions in seconds for the different Reference and Subject time points.

Although a single measurement time point could be taken, to be able to study the different MST zones, we placed Subject and Reference time points in four different zones (Figure 4). Usually, the mean of a particular zone is taken in a period spanning 0.5 to 1.0 s long [5,6,26,27,68].

The T-jump establishes the heat gradient and thus consists of a fast increase in temperature change at the illumination site; based on experiential studies, the full heat gradient takes one second to establish [26]. The TJ Subject point was therefore placed to partially overlap and capture the end of the TJ effect (1 s) at 5.94 s and span 0.5 s; its Reference point began at 2.44 s during the time the IR laser is still off, lasting 1 s (Figure 4).

Thermophoresis is the diffusion process that begins after the heat gradient is established [6,26]. During this time, there is a net migration of molecules out of the center of the illumination spot until a new steady state is reached, normally 30 to 60 s later [68]. Thus, in theory, any point after the temperature gradient is created should be valid as a Reference point. To completely avoid the T-jump, we set the TmF Reference point to begin at 6.8 s, lasting 1 s. Conceptually, the thermophoretic movement should be constant until the new steady state is reached [26]; however, we did not see any studies that actually looked at this. As mentioned earlier, the actual placement of the Subject and Reference time points is somewhat arbitrary, so we decided to test how the TmF values (as K_D_) varied with respect to the length of the TmF measurement; therefore, we moved the Subject time point in 1 s gapped steps across the TmF time trace (Figure 4).

The Inv. T-jump seems to also be completed in 1 s [26]. Its Reference time point was placed right before the IR laser was shut off and the Subject time point set to also include the actual end of the phase (Figure 4).

The BdF, therefore, commences after the temperature gradient collapse, whereby we added a small gap before setting the Reference point; the Subject time point was set before the measurement ended, each only a half a second long (Figure 4).

Three analysis software programs were evaluated: two from Nanotemper: NT Analysis Software and MO Affinity Analysis v2.3; and one from the Brautigam group: Python-based AnaLyser of MicroScale Thermophoresis, a.k.a. PALMIST [5]. All gave the same results when loading the data and choosing the same Reference and Subject time points. We chose to use PALMIST for our entire analysis as when combined with GUSSI [70], it allowed us to comfortably pool and analyze the replicates, including the plotting of fits and their variance.

#### 2.4.2. Comparison of the K_D_ from Different MST Zones

**Figure 5:**
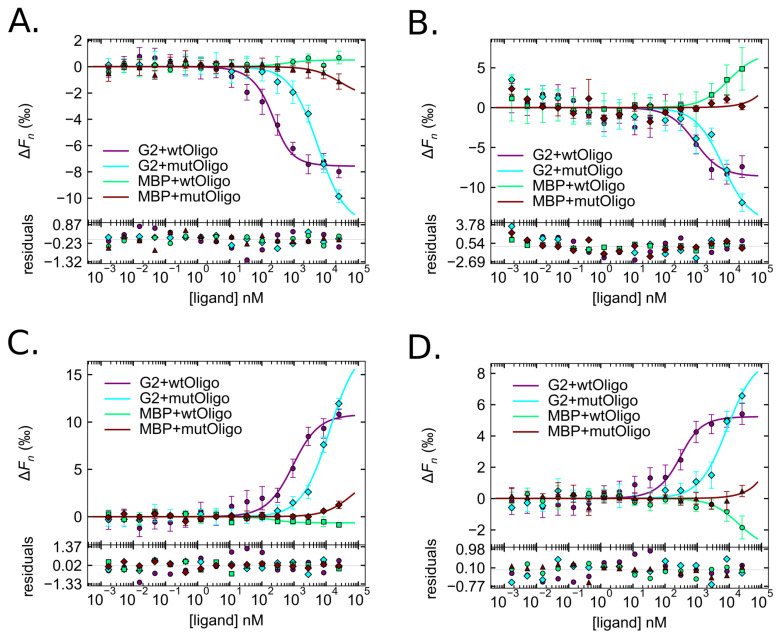
His–MBP–GARP2 interacts with DNA in a sequence-specific manner as shown by MST Interaction assays. Binding Curves of His–MBP–GARP2 (G2) or His–MBP (MBP) proteins to either wild-type (wtOligo) or mutated (mutOligo) CRM sites in a fragment of the *ARR5* promoter. The change in thermophoresis caused by the titration of the ligand (WT or mut Oligos) is expressed as the change in the normalized fluorescence (ΔF_n_). ΔF_n_ is defined as F_SUB_/F_REF_ ×1000 with F_REF_ the average Reference fluorescence value and F_SUB_ the average Subject fluorescence. *n* = 3 with ± standard deviation. The bottom panel depicts the residuals between the data and the fit line. (**A**) T-jump region; (**B**) Thermophoresis region 4_10_/3; (**C**) Inv. T-jump region; (**D**) Back diffusion region. Binding curves were fitted according to a one-to-one binding model using the PALMIST 1.4.4 software. Both His–MBP–GARP2 and His–MBP proteins were fluorescently labeled with RED-tris-NTA dye.

After protein purification and quantification of fluorescently labeled His–MBP–GARP2 (GARP2) and His–MBP (MBP), each protein was challenged against different wtOligo and mutOligo concentrations that went over eight orders of magnitude. Before an MST analysis was conducted, the samples were allowed to go to equilibrium [5,26]. The binding curves for each of the four MST zones were computed and are shown in Figure 5. 

**Figure 6:**
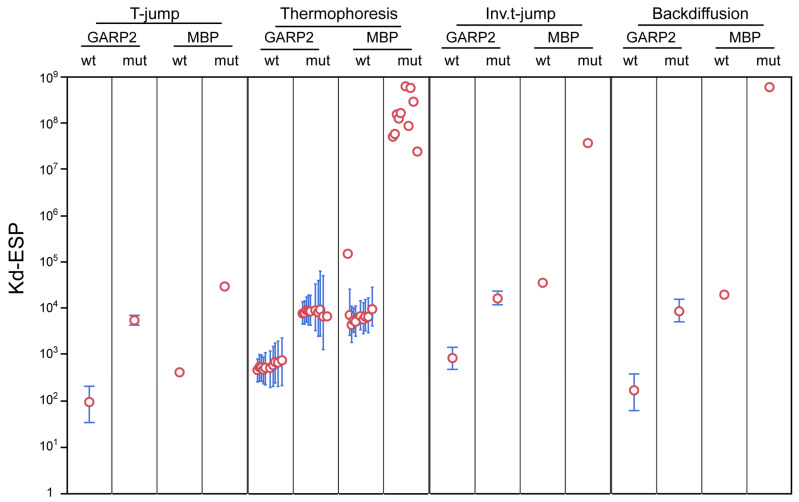
MST Dissociation constants K_D_ (nM) derived from MST binding curves for His–MBP–GARP2 and His–MBP interaction to DNA. K_D_ values are calculated from binding curves derived from different Regions of the thermophoretic time traces of His–MBP–GARP2 (GARP2) and His–MBP (MBP). These Regions include the T-jump region (Region 1–2); Thermophoresis regions (Regions 3–4_1_ to 4_10_); Inv. T-jump region (Region 5–6); and back diffusion region (Region 7–8). The 10 K_D_ values corresponding to the 10 different Thermophoresis regions (Regions 3–4_1_ to 4_10_) are in order; the first K_D_ value corresponds to Region 3–4_1_ and the last K_D_ value to Region 3–4_10_. K_D_ values were calculated according to a one-to-one binding model using the PALMIST 1.4.4 software. The data represent the different K_D_ values with their corresponding upper and lower bounds calculated with the error surface projection (ESP) method (0.683 confidence level). Those K_D_ that lack an interval value are unbound (see Table 1). The wild-type oligo (wt) comprises a fragment of the *ARR5* promoter region containing four (A/G)GAT(T/C) binding sites. The mutated oligo (mut) has these four binding sites mutated.

Across all metrics, GARP2 migrated out of the illuminated volume quicker with wtOligo at lower oligo concentrations compared to all other combinations. GARP2 with mutOligo also showed altered migrations, but these only occurred at the higher oligo concentrations. No significant migration differences were found for MBP with wtOligo or mutOligo. The K_D_ was calculated using the error surface projection (ESP) method at the 0.683 confidence level [5] and is given, along with ESP limits and confidence intervals σ, for all curves in Table 1 and summarized as a graphical cross comparison in Figure 6. 

Inspection of the derived K_D_s allows one to determine what constitutes an informative value. Across all metrics, GARP2 with wtOligo was the only curve that went to saturation, having K_D_s that ranged from 90 to 800 nM, well within the range of biologically meaningful values. We observe that GARP2 appeared to show weak binding with the mutOligo based on the bind curve appearance, but the binding curves do not go to saturation for any metric, and as such yield very high K_D_s, typically in the thousands (range ~5000 to ~14,000 nM). This range is similar to that also obtained for MBP with wtOligo, which we know does not bind to DNA based on our previous experiments. Furthermore, some of the MBP with wtOligo K_D_s and all of the MBP with mutOligo K_D_s were exorbitantly large and unbounded (Table 1, Figure 6), indicating that they are meaningless. Taken together, only the GARP2 with wtOligo binding curves show real and relevant binding for all of the thermophoretic metrics.

After analyzing the various MST zones as a whole, a detailed study for each thermophoretic metric was conducted to evaluate the different KD values obtained for each MST region. The KD values at the T-jump zone, showed that GARP2 bound to the wtOligo with a KD of 93 ± 40 nM [ESP bounds 34–204] while the KD for the mutOligo was much higher (5422 ± 662 nM [4272–6929]). As expected from our previous data, MBP neither bound to wt nor mutOligos. For the MBP/wtOligo interaction, the KD was 406 nM, but is unbounded, indicating that it does not represent a real KD; un-bounded values indicate that no function minimum could be found and thus that the fit is unreliable. The MBP/mutOligo KD was extremely high (29,278 nM) and also un-bounded. Based on these results, we demonstrated again that GARP2 binds to the wtOligo, and that the T-jump carries information about the binding of GARP2 to DNA and represents a real value.

At the thermophoresis zone, the KD for the GARP2/wtOligo interaction stayed quite constant throughout the different intervals, with values that slightly increased at longer intervals and that ranged from 456 nM at 41 to 738 nM at 410. All of the ESP bounds have a lower value around 200 but an increasing upper bound that is proportional to the standard deviation (SD) error. Interval 41 still might be too close to the T-jump signal and thus might not represent a reliable signal. The intervals 42 to 46 gave steady KD values around a mean value of 505 nM [ESP bounds 193–1174], with SDs that also stayed constant and around 200 nM. At intervals 47 to 410, KD and SD values became progressively higher. This suggests that choosing an interval zone too close to the end of the measurement cycle where one is very close to, if not inside of, the steady state might not be reasonable. It is important to note that although the KD for the GARP2/wtOligo interaction obtained in the thermophoretic zone is higher than that of the T-jump, both are well within the range of biologically meaningful values. In contrast, the KD values for the GARP2/mutOligo interaction were, in most cases, over 16 times higher than those for the GARP2/wtOligo couple, and the binding curves of this inter-action never reached saturation (Figure 5B and Figure 6 and Table 1). In conclusion, GARP2 might bind the mutOligo to some extent but in a much less efficient way than the wtOligo. The KD values for the MBP/wtOligo and mutOligo interactions were much higher and often unbounded, indicating again that the MBP does not bind DNA.

The Inv. T-jump involves the collapse of the temperature gradient, and in that re-spect, it is the inverse of the T-jump [26]. Here, only the GARP2/wtOligo combination had a reasonable KD and SD (826 ± 222 nM), very similar to that observed in the 410 thermophoresis zone, implying that the removal of the temperature gradient does not have the same effect as the application of one. All other protein–DNA combinations had exorbitantly large values and/or were unbounded.

Finally, the back diffusion zone consists of the recovery of the fluorescently labeled molecules into the observation point. BdF is reported to be dictated by the pure mass diffusion of molecules as they relax though the spatial gradient and should be de-pendent on the diffusion velocity as a direct function of size [26]. Much like the Inv. T-jump, the only reliable BdF value is that of the GARP2/wtOligo pair: 166 ± 74 nM. This KD is most similar to that of the T-jump.

In conclusion, at all thermophoretic metrics, only GARP2/wtOligo pairs had binding curves that went to saturation and KDs within the range of biologically mean-ingful values. A steady thermophoresis KD was observed, but only between the 42 to 46 intervals (~505 ± ~232 nM). Thermophoresis time points after this led to a steady increase in KD along with increasing uncertainty. The T-jump (93 ± 40 nM) and BdF (166 ± 74 nM) had the lowest and roughly comparable KDs, which are 4 to 5 times lower than that of the thermophoresis KD (~505 ± ~232 nM). The Inv. T-jump KD (823 ± 222 nM) matched the final 410 thermophoretic time point KD such that, at least for our experiment, the Inv. T-jump did not have the same thermal effects as the T-jump on the fluorophores within the illumination spot.

### 2.5. Structural Modeling of GARP2

**Figure 7:**
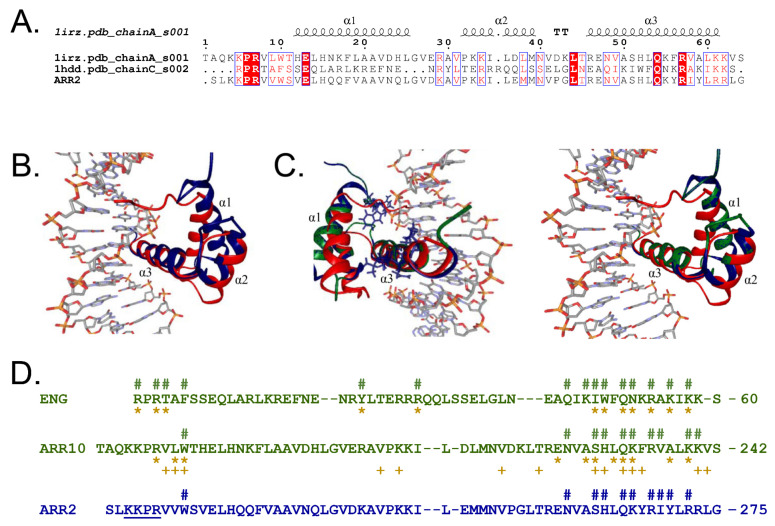
Structural modeling of ARR2 DNA-binding GARP domain. (**A**) The GARP domain of ARR2 (ARR2) structurally aligned to the crystal structure of ENG (1hdd.pdb_chainC_s002) and NMR structure of ARR10 GARP domain (1irz.pdb_chainA_s001). α-helices (α1, α2 and α3) are shown as squiggles and strict β-turns are shown as **TT**. Red box with white characters indicates strict identity. Red characters signify similarity in group; blue-framed characters signify similarity across groups. See ESPript [74] for more information. (**B**) Schematic model of ARR10 GARP domain (PDB id: 1IRZ) (blue) mapped onto ENG (PDB id: 1HDD) (red) interacting with DNA. (**C**) Schematic model of ARR10 GARP domain (PDB id: 1IRZ) (blue) and the modeled structure of ARR2 GARP domain (green), both mapped onto ENG (PDB id: 1HDD) (red) interacting with DNA. Amino acids the α3-helix and the N-terminal arm of ARR10 GARP domain shown in stick model when forming contact with DNA as described by Hosada and co-workers [33]. (**D**) Sequence alignment of the ARR2 and ARR10 GARP domains to ENG with emphasis on the DNA contact residues (#) predicted by our modeling for GARP2 (blue), GARP10, and ENG (top lines, green) compared to GARP10 contact residues of Hosoda et al. [33], Molecular Structure of the GARP Family of Plant Myb-Related DNA Binding Motifs of the Arabidopsis Response Regulators, The Plant Cell, 2002, 14, 9, pp.2022, adapted by permission of Oxford University Press; (lower lines, gold) derived from intermolecular NOEs (*) and NMR chemical shift perturbations (+), as well ENG contact residues given by Kissinger and co-workers [38] (lower lines, gold) derived from the crystallization of the ENG–DNA complex (*). Putative NLS for GARP2 is underlined.

Currently, there is a large shortage in the structural knowledge of B-type ARRs since only the three-dimensional structure of GARP10 has been solved experimentally (PDB id: 1IRZ) [33]. GARP10’s structure is similar to that of the ENG homeodomain protein and they both bind to DNA in an analogous manner [33,38]. GARP10 and ENG are composed of a helix–turn–helix motif that includes three α-helices (α1, α2 and α3) spaced by type I and II β-turns, respectively (Figure 7A), representing a common DNA-recognition unit present in numerous DNA-binding proteins [33,71,72,73]. The GARP10 motif recognizes DNA’s major groove by positively charged residues in its α3-helix, whereby the N-terminus possesses a flexible arm that makes contact to the minor groove [33].

When comparing GARP2, GARP10, and ENG secondary structures, all three proteins share a high degree of sequence similarity, and although the HTH motif is conserved in all three proteins, identical amino acids between GARP2 and GARP10 to ENG are restricted to the α_3_-helix and the N-terminus of the protein (Figure 7A).

To elucidate GARP2’s 3D-protein structure and which amino acids are responsible for DNA contact, we created an in silico model of a GARP2–DNA complex. To predict the structure of this complex, we first modeled GARP2 using the GARP10/GARP2 sequence alignment presented by Hosoda and colleagues [33] and the NMR structure of GARP10 (PDB id: 1IRZ) [33]. By using GARP10 NMR protein structure as a structural template for homology modeling, we could create a three-dimensional structural model for the GARP2 protein (Figure S1). Then, we mapped these two GARP structures to that of the ENG–DNA complex (PDB id: 1HDD, Chain C) [38] using Cα backbone atoms (Figure 7B,C). The structural superimposition of GARP10 (PDB id: 1IRZ, first model) on the ENG protein present in the ENG–DNA complex (PDB id: 1HDD, chain C) (Figure 7B) indicates that both proteins seem to share very high structural similarities (RMSD ≤ 2.6 Å). It also creates, for the first time, a GARP10–DNA complex (Figure S2). This model was further used to identify contacts between the transcription factor and the DNA. The superimposition of the three transcription factors—ENG, GARP10, and GARP2—allowed us to map potential DNA contact sites in the GARP2 domain (Figure S3). Since GARP10 and ENG seem to bind to the DNA through the α3-helix and the N-terminal arm of the protein [33,38], and since GARP2 was modeled using the three-dimensional structure of GARP10, it is expected that GARP2 also contacts DNA the same way.

As seen in Figure 7C, the α3-helix mapped extremely well to all three proteins, and it seems to be located in the major groove of the DNA, forming sequence specific interactions; the structural similarities are followed in the α_2_-helix, with the α_1_-helix realigning at the N-terminal tail of the domain. The GARP2 homology modeling yielded an RMSD of 2.6 Å for all Cα atoms between ENG (1HDD, chain C) and GARP2, indicating that the GARP2 modeling also produced a very good model.

Regarding how these domains bind to DNA, we were restricted to the information of the DNA/ENG crystal structure since the available NMR of GARP10 was performed without DNA. Intermolecular NOEs and NMR chemical shift perturbations studies predict the way the DNA–GARP10 complex could be formed, showing that GARP10 residues involved in DNA binding seem to highly correlate with those involved in the formation of the ENG–DNA complex [33,38] (Figure 7D).

To understand how the GARP2–DNA complex could be formed, we used our in silico model of a GARP2–DNA complex to inspect which GARP2 amino acids might likely be located close to DNA. First of all, we studied how well we could identify the DNA contact sites established for the ENG–DNA complex (PDB id: 1HDD, Chain C) [38]. Here, we could identify 15 amino acids of ENG that are close to DNA atoms (Figure 7D) that coincide with those established by Kissinger and colleagues [38]. When we compare our results to the contacts predicted by Hosoda and colleagues [33], we identify three more (F8, Q44, K46). Hosoda and co-workers [33] predicted the GARP10 amino acids that potentially contact DNA via intermolecular NOEs and NMR chemical shift perturbations studies. Hosoda and co-workers [33] identified three more amino acids in the α_3_-helix of GARP10 that were predicted to contact DNA (A228, L231, and V241) that did not appear in our prediction. In the N-terminal arm, we could only identify one putative contact (W188) compared to the total of four predicted in 1IRZ [33]. The reason for this could be that the N-terminal arm is flexible and when the protein structure is modeled without DNA present, it might not be located in the correct position. Taken together, our modeling also produced a very good prediction of the DNA contact sites in ENG and GARP10.

Based on our model, GARP2 amino acids N259, S262, H263, Q265, K266, R268, I269, Y270, and R272 in the α_3_-helix of GARP2 can putatively form direct interactions with DNA (Figure 7D). In addition, our model also predicted that W221 in the N-terminal arm of GARP2 binds DNA. It is interesting to note that from the nine amino acids predicted to contact DNA in α_3_-helix of GARP2, seven of them (S262, H263, Q265, K266, R268, I269, and R272) coincide with amino acids that bind DNA in ENG and/or are predicted to contact DNA in GARP10 [33,38]. GARP2 W221 amino acid that is predicted to contact DNA through the N-terminal arm also coincides with W188 in the ARR10 that was predicted to contact DNA in both intermolecular NOEs and NMR chemical shift perturbations studies [33].

In conclusion, the sequence alignments and secondary structure information indicates that the GARP domains of ARR2 and ARR10 are very similar and could bind DNA in the same way. We could map them independently onto the structurally similar protein ENG and show that the models form contacts with DNA that match NMR experiments. This correlates well with the fact that both ARR2 and ARR10 proteins generally bind to the same GARP motifs in vitro and in vivo [33,45]. Based on structural models, we postulate that ARR2 binds to DNA principally through the α_3_-helix of its GARP domain and possibly through the N-terminal tail as well.

### 2.6. Mutations in the GARP2 Domain Affect ARR2’s Ability to Activate Transcription

We have established via in silico modeling of the GARP2–DNA complex that amino acids in the α_3_-helix and the N-terminal tail of the GARP2 domain are likely responsible for contacting DNA and thus indispensable for ARR2 to function as a transcriptional activator. To study this, we created an ARR2 mutant (ARR2^mG2^), where some of this putative DNA binding residues were mutated to Ala, namely, amino acid W221 responsible for contacting DNA through the N-terminal tail of the GARP2 domain and amino acids HLQ in positions 263 to 265 in the α_3_-helix region.

**Figure 8:**
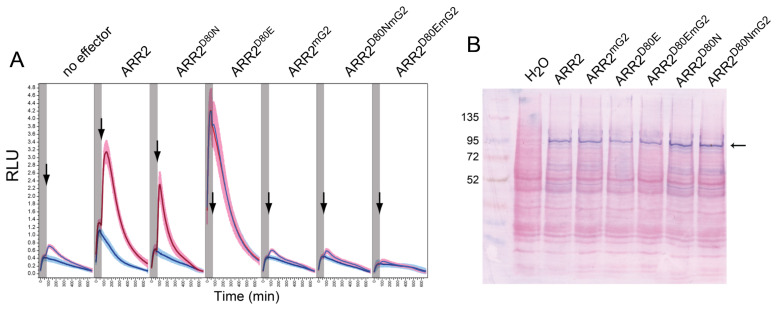
Mutations of the GARP domain in putative sites that directly contact DNA results in a reduction of ARR2 transcriptional activation ability. (**A**) Transactivation assay of the A-fragment of the *ARR5* promoter fused to Luciferase (*ARR5p::LUCm*^3^) with ARR2, ARR2^D80N^, ARR2^D80E^, ARR2^mG2^, ARR2^D80NmG2^, ARR2^D80EmG2^ as effectors after cytokinin (red) or mock (blue) treatments. The grey bar represents the flash measurement prior to cytokinin addition. The black arrow marks the time point of hormone application. Light emission is given as relative LUC activity (RLU). The lighter area around the curves represents the standard error calculated from four technical replicates. (**B**) Immunoblot using a HA-specific antibody of crude extracts from protoplasts expressing ARR2 and ARR2 mutant proteins and Ponceau staining. H_2_O: control—water-transfected protoplasts.

We carried out transactivation assays as described by Wallmeroth and colleagues [55] using the B-fragment of the *ARR5* promoter fused to luciferase (*ARR5p::LUCm*^3^) as the reporter gene and ARR2 and ARR2^mG2^ as effectors (Figure 8A). As already described, cytokinin treatment promotes reporter gene activation when wild-type ARR2 is used as an effector. In contrast, no transcriptional activity was observed when ARR2^mG2^ was overexpressed, thus defining the mutated GARP residues as essential in ARR2 function.

We also studied how ARR2 function was affected when mutations in the GARP domain were combined with a mutated D80 residue. The conserved aspartate in position 80 (D80) is responsible for ARR2 activation via phosphorylation, and mutations of this residue have been shown to result in constitutively active (D80E) or partially inactive (D80N) response regulators [47,56,59]. We created ARR2 higher-order mutants containing the D80E and the D80N mutations in combination with the GARP mutations (ARR2^D80E/mG2^ and ARR2^D80N/mG2^). In transactivation assays, ARR2^D80E^ overexpression resulted in reporter gene activation in a cytokinin-independent manner, as reported previously (Figure 8A) [55]. Interestingly, a mutated GARP domain in the ARR2^D80E^ background eliminated the ability of the response regulator to activate transcription, as observed when ARR2^D80E/mG2^ was overexpressed under mock and cytokinin treatments (Figure 8A). This suggests that the activation the response regulator via its conserved aspartate residue has no effect on the response regulator’s activity if the response regulator is not able to bind DNA through its GARP domain. In the case of the ARR2^D80N^ mutant, our data corroborated that the D80N mutation results in a partially inactive response regulator both under mock and cytokinin treatments (Figure 8A), as previously observed [53,55]. Interestingly, the introduced mutations in the GARP domain completely blocked its function when ARR2^D80N/mG2^ was overexpressed (Figure 8A).

Differences in transactivation levels were not due to differential protein expression. As shown in Figure 8B, all ARR2 proteins were expressed equally.

To summarize, similar to ARR10, the ARR2 GARP domain is essential for DNA binding, and specific residues within this domain, both in the N-terminal tail and the α_3_-helix region, are critical for contacting DNA.

## 3. Discussion

The isolated GARP10 DNA binding domain was first characterized in 2002 by Hosoda and co-workers [33]. Although diverse in vivo and in vitro studies have been conducted on B-type ARRs since then, to our knowledge, none have had a look at the dissociation constant of a GARP domain beyond ARR10. We show that GARP2 binds to a native stretch of DNA from the *ARR5* promoter via its known conserved binding site using three wet-lab methods and one in silico. We have estimated the K_D_ of the GARP domain of ARR2 interaction to DNA using MST and confirmed it by binding by DPI-ELISA and FCS. In addition, we structurally modeled GARP2 in silico to determine which amino acids most likely make direct contact to DNA. Finally, we conducted transactivation assays which showed that GARP2 can activate transcription of the *ARR5p::LUCm*^3^ reporter gene, while a mutated version of GARP2, with mutations in several amino acids that putatively contact DNA, could not.

The *ARR5* promoter is a target of B-type ARRs [55,75]. The ARR5 protein is involved in ABA and drought stress [76] and also negatively regulates the TCS [77]. Within the *ARR5* promoter, there are many CRMs that might work synergistically, as we showed that truncating the promoter led only to a reduction and not to a loss of responsivity to ARR2. For our GARP2–DNA interaction studies, we chose an *ARR5* promoter fragment that contains three CRMs and one ECRM (termed wtOligo) and generated a mutated version where the highly conserved nucleotides *TC* of the CRM motive were replaced with *AG* (termed mutOligo).

ARR2 is predicted to have at least three NLSs [45,57,78], and we probably identified at least one of them since our GARP2 domain is nuclear-localized. GARP2 is thus a multifunctional domain just like GARP10 [33], carrying an NLS as well as binding to DNA. Our DPI-ELISA experiment demonstrated that GARP2 could bind to the natural CRM cluster of the *ARR5* promoter and that this was dependent on the conserved CRM motifs since GARP2 did not bind to oligos containing mutated CRMs. The DPI-ELISA was thus greatly informative, indicating that our cloned GARP2 was functional and that the CMR cluster was indeed a target of the GARP2 domain. In addition, because the DPI-ELISA was performed using crude protein extracts, the GARP2/wtOligo had to be strong enough to outcompete any other proteins in the sample that could also bind to the *ARR5* promoter fragment. To avoid the effect of additional *ARR5* promoter interactors and to test the GARP2/wtOligo interaction with an additional method, we proceeded to examine the DNA binding affinity of purified GARP2 with FCS that would allow us to measure the average diffusion coefficient of the DNA–protein complex. Consistent with previous results, we observed that GARP2 binds to the wild-type but not to the mutated oligo, affecting the diffusion coefficient of the wtOligo in a GARP2 concentration dependent manner. Furthermore, we carried out a competition experiment where, at a set concentration of GARP2 protein, the diffusion time of the labeled wtOligo was affected by an excess of unlabeled wtOligo; corroborating again that the GARP2 domain binds to specific sites of the *ARR5* promoter in a reversible manner.

To complete the GARP2–DNA interaction studies, we turned to MST, which allowed us not only to corroborate the binding of the GARP2 domain to specific sites of the *ARR5* promoter but also to calculate the K_D_ of this interaction. MST is an optimal method by which to study bi-molecular interactions for many reasons; theoretically, it avoids specific surface trapping artifacts and limitations due to molecular weight ratios as in SPR or FCS [6,26,27,79]. In addition, it is a very sensitive technique since thermophoretic events are affected by minor variations in the molecule–solvent interface, and even tiny changes in the conformation, charge, or size of the molecule can have an impact on its thermophoretic movement [6,26].

In MST measurements, one must place both a Reference/cold and Subject/hot region in order to identify the amount of bound and unbound molecules. Placement of these two points seems to be rather arbitrary [5,6,26] and is limited to a visual trace curve inspection or a cross-reference with another, independent K_D_ study for justification of the chosen points. To avoid losing any information due to subjective placing of these two points, we took an unbiased look at all of the thermophoretic regions and their effects on the K_D_ prediction. We observed that GARP2 bound to wtOligo specifically under all thermophoretic effects. Our findings also suggest that GARP2 might also bind, albeit in a much weaker manner, to mutOligo. The MBP control protein did not bind DNA, although some molecular crowding effects could be seen in the binding curves. Molecular crowding can make an MST prediction drastically unreliable [3,5]. In addition, DNA oligomers are said to be prone to convection due to the strong thermophoresis of DNA [80]. We think we see some of these effects in our MBP control at very high oligo concentrations; in any case, these binding curves never reached saturation and yielded very high senseless K_D_s.

The temperature jump is a localized event in which fluorescence changes are primarily due to temperature dependent changes to the fluorophore, affecting its absorption, lifetime, or even quantum yield parameters. Nevertheless, a binding event may or may not affect T-jump [26]. In our case, the T-jump zone clearly had a saturated binding curve and provided a K_D_ of 93 nM for the GARP2/wtOligo interaction. In contrast, the GARP2/mutOligo interaction yielded a K_D_ of 5423 nM, approximately 58 times weaker. This is similar to studies of other TFs which showed that single-point mutations in the DNA motif can lead to large increases in K_D_s from 44 to 2000 times weaker [79].

In our experiments, the K_D_s obtained for both the T-jump and the back diffusion are very similar. BdF is said to only be the pure mass diffusion of molecules. After the collapse of the temperature gradient, the spatial concentration gradient starts to relax, and the time needed for the homogenous distribution of molecules is dependent on the diffusion velocity, which is in turn dependent on the molecular size [26].

Oddly enough, the K_D_ for the TmF zone was actually the most complicated to determine and to interpret. Fortunately, however, by using our time-scanning approach to TmF, we believe we have captured the entire range of this zone and were able to calculate and compare the different K_D_ values for the complete zone. TmF captures a global change on the labeled molecule and is not locally dependent on the dye. This means that changes in the charge, size, and solvent shell due to interaction can all contribute to a difference in the thermophoretic movement of a molecule. Furthermore, our choice of the different TmF intervals to yield different K_D_s is also validated by the fact that thermophoresis does not have to reach a steady-state level to yield a dissociation constant [26]. In our experiments, the most stable thermophoretic movement that correlated with constant K_D_ values (~505 nM) occurred between intervals 4_2_ to 4_6_. This timeframe is congruent with the interval chosen for several publications [80,81,82,83]. In contrast, K_D_ values increased at later intervals (from 4_7_ to 4_10_). Thus, measuring at the end of the steady state curves is likely not capturing the thermophoretic motion. Finally, the Inv. T-jump K_D_ (823 nM) matched the final 4_10_ TmF time point K_D_.

Hosoda and colleagues [33] studied the DNA binding ability of ARR10 GARP domain by gel-shift assays and SPR. They concluded that the optimal recognition sequence of GARP10 is 5′-AGATT-3′. They also quantified the binding affinity of GARP10 to a synthetic 12-bp oligonucleotide that contained a 5′-AGATT-3′ copy to a K_D_ of 600 nM [33]. It is tempting to match our MST K_D_s to theirs since the values are extremely close; the K_D_ for the GARP2/wtOligo interaction is ~505 nM for the TmF zone. However, we should keep in mind that different methods and DNA sequences have been used in both experiments. In any case, we can conclude that GARP2 strongly binds to the *ARR5* promoter sequence trough the CRM’s sequences. The K_D_ of this interaction is very similar to that of GARP10 and within a biologically meaningful value that ranges from 93nM at the T-jump to 826 nM at the Inv. T-jump.

In agreement with these findings, our modeling analysis also indicates that the GARP10 and GARP2 domains are very similar, and both seem to bind DNA in the same way; most probably using specific amino acids in the α_3_-helix and N-terminal tail of the GARP domain to directly contact DNA. The experiments carried out with the mutated GARP2 also validate the in silico data since they point out specific residues that are most probably responsible for directly contacting DNA and which are thus required for the GARP2 function as a transcriptional activator. We tested this with an in vivo transactivation assay in which some of the predicted DNA contact residues of GARP2 were mutated. Provocatively, all ARR2 variants did not activate the promoter–reporter when GARP2 was mutated, suggesting that DNA binding is likely required for their transactivation capacity.

There are over fifty genes in Arabidopsis that encode a GARP domain [34] and although similar, they are distinct enough to bind to different elements [34,84]. Previous in vitro and in vivo studies have repeatedly demonstrated that the subfamily-1 of B-type ARRs (ARR1, 2, 10, 12, 11, 14, and 18) bind to very similar core DNA elements with variable flanking nucleotides [45,51,84,85,86], all of which revolve around a 5′-(A/G)GAT-3′ core. Xie and colleagues [85] demonstrated that this variability is drastically reduced when tissue is exogenously treated with cytokinin converging on a consensus sequence 5′-AGAT(A/T/C)(T/C)-3′ for ARR1, 10 and 12 [33].

We showed that GARP2 binds to DNA using three different but complementary wet-lab techniques. Each method could clearly demonstrate the binding of GARP2 to consensus DNA motifs, albeit to different degrees. qDPI-ELISA that used bacterial crude protein extracts effectively revealed specific interactions compared to the controls, demonstrating that GARP2 does indeed recognize CRM elements as expected. Affinity purification, however, would likely increase the quantitative range of this method, making it even more sensitive to lower affinity binding interactions.

FCS was then attempted, and in our hands, it worked best with purified GARP2 protein. FCS not only confirmed the qDPI-ELISA results but also allowed us to determine how the diffusion constant of the wtOligo changed when bound to GARP2 as a complex. FCS also enabled us to define that the GARP2/wtOligo interaction occurs in a reversible manner. This indicates that FCS is also an excellent candidate through which to study allosteric factors that might affect DNA–protein complexes or even protein–protein complexes, albeit once the factors are purified.

While both methods were effective in confirming the previous interaction of the GARP2 domain with its DNA consensus target, neither was suitable to give us an insight into its possible interaction strength. For this we turned to MST, where we also used purified protein in order to avoid non-specific background competition. MST again confirmed specific binding of GARP2 to CRM motifs and further allowed us to compute Kds from multiple experimental conditions and under different thermophoretic effects. Interestingly, informative Kds were obtained for all four measurement zones when GARP2 was bound to its cognate DNA. Thus, each zone does indeed carry utilitarian information about molecular interactions, making it difficult to choose a particular one.

Our structural modeling suggested that DNA binding likely occurs with same conserved residues as GARP10, and it probably does so with a comparable structure. While only predictive, this method still provided a much deeper view of the GARP2 domain that went beyond those obtained via secondary structure predictions since it allowed us to underline the putative amino acids involved in direct DNA contact. Our study is the first to mutagenize these putative DNA contact amino acid residues of a B-type ARR and show their importance in vivo, demonstrating that the GARP2 is not only able to bind specific DNA sequences but that these residues are essential for ARR2, in its full-length context, to act as a transcriptional activator of its target genes. Taken all together, and considering previous work in the field, it is most probable that all of the subfamily-1 B-type ARRs use the same amino acid residues to contact DNA and do so with similar binding affinities.

In summary, each method yielded valuable insight about the GARP2–DNA complex formation, giving complementary yet distinct information based on how each method works. By presenting a direct comparison of these different methods using the same DNA–protein pairs, researchers can choose experimental approaches that are best suited to them, considering how much time and resources they have available. These methods should be applicable for any protein–DNA pair, and it will be interesting to test in future research if the interaction of full-length B-type RRs with their cognate DNA is also possible.

## 4. Methods

### 4.1. Cloning of GARP_ARR2_

GARP2 was cloned into into pENTR™/D-TOPO^®^ with forward primer 5′-*caccatgtcatcgagtttaaagaaac*-3′ and reverse primer without 5′-*tccaagccgtctcagatatatccg*-3′ followed by Gateway recombination into *pABind::GOI::GFP* [53] for expression in planta and into *pET-Dest42-GFP* (Thermo Fisher Scientific, Karlsruhe, BW, Germany) for expression in bacteria. *GARP2* was classically cloned via BamHI/HindIII into *pET-His8–MBP* [87] after PCR amplification with forward primer 5′-*gagaggatcctcatcgagtttaaagaaaccac*-3′ and reverse primer 5′-gagaaagc*ttatccaagccgtctcagatat*-3′. Insertions were checked by restriction digestion followed by sequencing of the insert and linker region.

### 4.2. Target DNA

The wtOligo (5′-ttaacAATCTcaaAGATTttgtAGATTgaaatacaAATCTtctct-3′) comprises a fragment of the *ARR5* promoter region containing four core GARP 5′-(A/G)GAT(T/C)-3′ binding sites (see main text). The mutOligo (5′-ttaacAAagTcaaActTTttgtActTTgaaatacaAAagTtctct-3′) has these four binding sites mutated. The forward and reverse stand were synthesized, and one strand was 5′ of the antisense strand, were coupled to biotin or Alexa-647 for DPI-ELISA or FCS, respectively, or unlabeled for MST experiments.

### 4.3. DPI ELISA

#### 4.3.1. Bacterial Growth, GFP Induction and Extraction

BL21(DE3)-RIL (Agilent, Waldbronn, Germany) cells were grown overnight on 37 °C in 5 mL LB media under selective conditions. The next day, 2 mL were transferred to 1L LB media with antibiotics. The culture was grown at 37 °C until OD_595_ 0.5. Protein expression was induced with 1 mM IPTG for 6 h on 37 °C. Cells were collected via centrifugation at 2200× *g* at 4 °C for 20 min. Cell pellets were rinsed in 50 mL ice cold Tris/NaCl (10 mM Tris-HCl (pH 7.5–8), 100 mM NaCl). After centrifugation (2200× *g*, 4 °C, 20 min), the cell pellets were resuspended in 4 mL DPI-Ex-buffer (4 mM HEPES (pH 7.5 KOH)), 100 mM KCl, 8% Glycerol (*v*/*v*), and Proteinase inhibitor (Roche Complete-EDTA); after the Bradford assay, 0.2% BSA (biotin free) and 1 mM DTT were added. Cells were lysed via sonication (Bandelin, Sonopuls HD2070 with MS72 Sonotrode) on ice 6 times for 15 s at 75% power ON and 15s OFF. Cell fragments were removed by centrifugation (2200× *g*, 20 min, 4 °C). Total protein amount was estimated via a Bradford assay. Cell extracts were either used right away or frozen at −20 °C. If frozen, samples were cleared at 14,000 rpm for 15 min at 4 °C.

#### 4.3.2. Preparation of Ds Oligos

ds oligos (10 µM in ddH_2_O) were mixed to a final concentration of 2 µM per oligo with annealing buffer (final concentration: 40 mM Tris/HCl (pH 7.5–8), 20 mM MgCl_2_, 50 mM NaCl). The oligos were incubated in a thermocycler for 3 min at 95 °C, followed by −1 °C/min temperature decrease to 28 °C.

#### 4.3.3. Immobilization of Ds Oligos to Plate

An amount of 5 pmol ds oligos were diluted in 27.5 µL TBS-T (20 mM Tris, 0.18 M NaCl (pH 7.5 with HCl), +0.1% Tween-20), pipetted to a well on a black 384 well streptavidin coated microtiter plate (Greiner 781997), and incubated for 1 h at 37 °C. After incubation the plate was washed 3 times with TBS-T. After washing the plate was blocked with 50 µL/well 1% BSA (biotin free) in TBS-T for 30 min at room temperature. The plate was finally washed 3 times with TBS-T before adding protein samples.

#### 4.3.4. Protein Binding and Fluorescence Measurement

An aliquot of protein was thawed on ice and cleared of aggregates by centrifugation. To balance the target protein, GFP emission was captured via aliquoting 15 µL to an uncoated, black 384 well plate and GFP measured with a BertholdTech TriStar2S plate reader (ex.485 nm, em. 535 nm, integration 100 ms, Xenon at 100%, PMT HV 1000V). The weakest GFP signal was set to 1 and a linear dilution factor was used for all other protein extracts; empty wells and buffer alone severed as reference controls for background noise. Thereafter, 15 µL + 15 µL TBS was loaded per well on the plate in triplicate. The plate was incubated for 1 h at RT (~25 °C) to allow binding; thereafter, the GFP signal was measured before and after each wash step. Each wash step was 2 times with 50 µL/well TBS-T followed by 2 times with 50 µL/well TBS. After the first measurement, the washing steps and measurement was repeated until the plate background level was reached.

#### 4.3.5. Quantification of GFP by Fluorescence

The eGFP concentration was determined by comparing its fluorescence intensity measured in a BertholdTech TriStar2S plate reader to a known concentration of FITC using the following formula:

$$\frac{\sum_{nm=478}^{492}0.33_{478-492}\times 76000_{FITC}\times 0.92_{FITC-QY}\cdot {Intensity}_{FITC-nm}}{\sum_{nm=478}^{492}0.25_{478-492}\times 56000_{eGFP}\times 0.6_{eGFP-QY}\cdot {Intensity}_{eGFP-nm}}.$$

### 4.4. Reporter-Gene Transfection Assays

Reporter gene assays were conducted and quantified exactly as described in Wallmeroth et al. [55] using the same *ARR5p::fLUCm*^3^ reporter. The mutations to the GARP domain W221A, H263A, L264A and Q265A were introduced by site-directed mutagenesis to wild-type ARR2 and the phosphate-accepting Aspartate ARR2 variants D80N and D80E [1] in pDONR vectors first, and were swapped in the pHBTL-3xHA vector by Gateway^®^ recombination. *ARR5p* truncations were classically cloned into *pBT8-LUCm*^3^ as described in [54] using reverse primer 5′-gatatggctgaggttttgcgtgccatggaaaa-3′ and fragment A (5′-aggatccgagttcgcggttcgacctaaact-3′), B (5′-aggatccatatattggtcgagtttagt-3′), D (5′-aggatccgctatcagacagacaaa-3′), and E (5′-aggatcctctccccatatcatatttttct-3′) via BamHI/NcoI.

#### Immunoblot Analysis

After an overnight incubation, the ten 30 µL protoplast transfection reactions were pooled, diluted 10-fold *v*/*v* with W5 (154 mM NaCl; 125 mM CaCl_2_; 5 mM KCl; 5 mM glucose) and pelleted at 50 g for 20 min, 4 °C. The cells were moved to a 2 mL Eppendorf, and repelleted at 100 g for 10 min, 4 °C, to remove any residual buffer. Approximately 100 µL of glass beads (∅ 0.1 mm) and 125 µL of Lyse and Load Buffer (50 mM Tris/HCl pH 6.8; 4% SDS; 8M Urea; 30% *v*/*v* glycerol; 0.1 M DTT; 0.005% Bromophenol blue) were added, vigorously vortexed for 2 min, incubated at 65 °C for 10 min, then centrifuged for 10 min 4 °C at 8000× *g*. The supernatant used for SDS-PAGE and wet immunoblotting onto PVDF. The HA epitope was detected with anti-HA mouse (Roche) antibody followed by an AP-conjugated anti-mouse (BioRad).

### 4.5. Nickel-NTA Purification

#### 4.5.1. Bacterial Growth and Protein Induction

An amount of 5 mL LB media containing the corresponding antibiotics for the expression plasmids in BL21(DE3)-RIL (Agilent, Waldbronn, Germany) was grown overnight shaking at 180 rpm at 37 °C. A total of 2 mL of this pre-culture was added to 300 mL TB media containing the corresponding antibiotics and grown for 4 h at 37 °C. The culture was cooled down on ice for 10 to 15 min and then induced by adding IPTG to a final concentration of 100 µM and grown for 20 h at 18 °C. The cells were collected at 2000 g for 5 min at 4 °C in a 50 mL falcon tube, yielding on average a pellet ~5 mL. Bacterial pellets were processed immediately or stored at −80 °C.

#### 4.5.2. Extraction

An amount of 15 mL of low-p buffer (25 mM phosphate buffer pH 7.5, 300 mM NaCl) was added to ~5 mL bacterial pellet, to which 18 µL β-mercaptoethanol and a pinch of lysozyme was added. The bacterial pellet was then dissolved for 30 min to 1 h at room-temperature on a spinning wheel. Then, 4–5 mL of glass beads (0.25–0.5 µm) were added and vortexed for 7 to 10 times, alternating 1 min vortexing followed by 1 min on water ice. Thereafter, 2 mL of 3 M NaCl was added to a final concentration of 600 mM, and the protein was pre-cleared via centrifugation at 3500 rpm for 30 min at 4 °C. The supernatant was cleared at 14,000× *g* for 30 to 60 min at 4 °C.

#### 4.5.3. Protein Purification

Ni-beads (~1.2 mL) were added to protein extracts (see above) in 50 mL Falcons (previously washed with ddH_2_O). To this mixture, 3 mL of 3M NaCl and 250 µL of 2M Imidazole pH 7.0 were added and filled up with low-p and closing the falcon so that there should be as less air as possible. Proteins were allowed to bind for 3–4 h on a spinning wheel at 4 °C. The mixture was then applied by gravity filtration to a standard chromatography column. Thereafter, it was washed from a minimum of 5 times the bead volume and up to 500 mL with low-p buffer with 10 mM imidazole. The protein was then eluted with low-p with 300 mM imidazole.

#### 4.5.4. Dialysis

Protein samples were dialyzed overnight in dialysis buffer (1/2 low-p buffer) in SnakeSkin^®^ dialysis tubing 10 kDa (Thermo Fisher Scientific, Karlsruhe, BW, Germany) then exchanged with fresh buffer for 3 more hours. If necessary, protein extracts were concentrated using VivaSpin 10 kDa centricons (Sartorius Stedim Biotech GmbH, Göttingen, Germany), whereby the sample was mixed between centrifugation steps.

#### 4.5.5. Determining Protein Concentration

The concentration of nickel-NTA-purified proteins was determined by measuring the absorbance at OD_595_ after reacting with Bradford reagent calibrated to a standard curve with BSA. A dilution factor was then calculated to bring the samples to 1 µM for experimental samples, unless indicated otherwise. Purified proteins were also inspected by Coomassie staining in poly-acrylamide gels.

### 4.6. FCS

FCS experiments were conducted as described previously in [88,89] with small modifications. Briefly, to assess His–MBP–GARP2 protein/oligonucleotide interaction, first, alexa647 fluorescently-labeled oligonucleotides were incubated in the presence or absence of non-labeled protein for 10 min at RT in PBS. The sample mixtures were measured in 8-well Lab-Tek chamber slides (NUNC). FCS measurements were performed at 25 °C using a ConfoCor3 module with attenuated excitation light from helium-neon laser (633 nm). To obtain autocorrelation curves, raw fluorescence fluctuation data were fitted to a 3D diffusion model as described in [88,89]. Irregular curves resulting from instability and distortion due to protein aggregates were excluded from the analysis. Unless stated otherwise, labeled oligos were used at 50 nM final concentration, unlabeled wtOligo at 500 nM, and His–MBP–GARP2 or His–MBP at 6 µM.

### 4.7. MST Preparation and Analysis

#### 4.7.1. Annealing Ds Oligos

Double-stranded oligos were formed by annealing 20 µL of sense oligo (100 µM in H_2_O) to 20 µL antisense oligo (100 µM in H_2_O) in PCR tubes and annealed to a final concentration of 50 µM in a thermal cycler for 3 min 95 °C and gradually decreased to 28 °C; thereafter, they were stored at 4 °C until use.

#### 4.7.2. Identifying the Optimal Capillary Type

An amount of 50 µL of His-Tag RED-tris-NTA dye was brought to 5 µM in PBS-T following the manufacturer’s instructions. Proteins were diluted in PBS-T to 200 nM. To label the protein; 100 µL (200 nM) protein was added to 100 µL (100 nM) NT dye in PBS-T, yielding a final protein concentration of 100 nM and dye concentration of 50 nM. After 30 min at room-temperature, the solution was cleared of aggregates by centrifugation for 10 min at 15,000× *g* at 4 °C. Standard and somewhat hydrophilic had less optimal curves; therefore, we choose MST Premium-Coated Cat# MO-K005 capillaries.

#### 4.7.3. Affinity Test and Labeling

**Figure 9:**
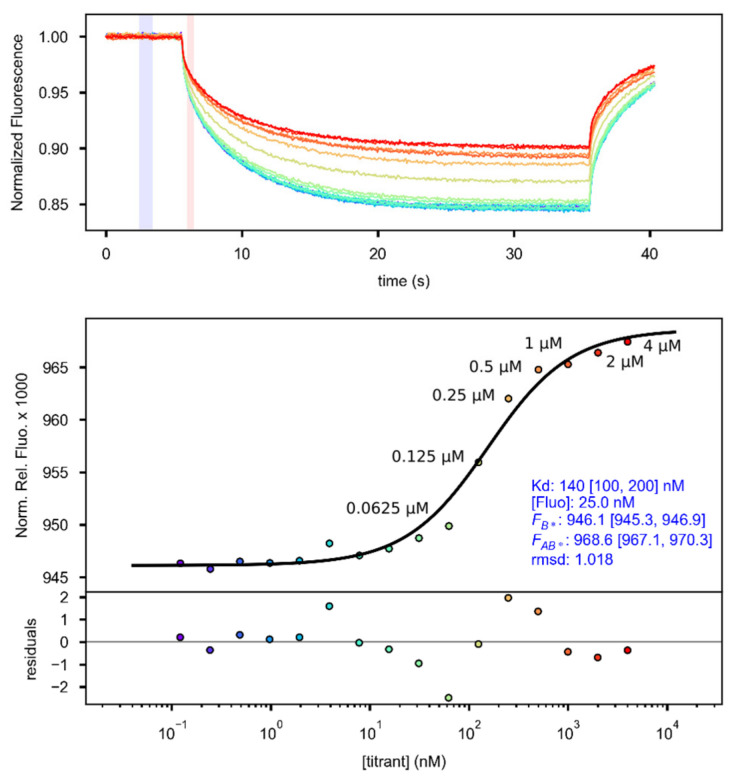
MST trace and binding curves to calculate RED-tris-NTA dye concentration for His-labeling. Scheme of the MST trace (**upper panel**) and binding curve (**lower panel**) of 25 nM RED-tris-NTA dye bound to different concentrations of His–MBP–GARP2 protein, correspondingly labeled and color tagged in both panels.

An amount of 200 µL of 50 nM His-labeling RED-tris-NTA dye was prepared in PBS-T, and purified His–MBP–GARP2 was brought to 4 µM in PBS-T. A total of 10 µL PBS-T was distributed to 16 hydrophobic capillary tubes 2 to 16. A total 20 µL of 4 µM His–MBP–GARP2 was added to Tube 1, from which 10 µL was moved to Tube 2, mixed well via pipetting, and 10 µL was moved to Tube 3, and so on till Tube 16, from which 10 µL was removed, leaving only 10 µL. To each Tube, 10 µL of 50 nM RED-tris-NTA was added, mixed, and incubated for 30 min at room-temperature. The final concentration of the RED-tris-NTA dye for each tube was 25 nM. These were then measured at 40% MST/40% LED power, which yielded a Kd (ESP) of 140 nM (Figure 9). Based on these results, 25 nM of RED-tris-NTA is able to maximally track up to 500 nM of protein at saturation, that is a 500/25 = 20 times labeling ratio. Since this was close to saturation, the next lower labeling ratio 250/25 = 10 times was chosen for all experimental samples, where we expect all of the dye to react with the protein, as recommend in the manual [67]. Therefore, for experimental samples we chose to label 1 µM of His-tagged purified proteins with 100 nM RED-tris-NTA in a two-step dilution scheme: 1 µM protein was mixed with 100 nM RED-tris-NTA 1:1, allowed to sit at room-temperature for 1 h, and then cleared of aggregates for 10 min at 4 °C, 15,000× *g*. The protein is then further diluted 1:1 with varying ds-oligos during an experiment, yielding a final protein concentration of 250 nM and 25 nM RED-tris-NTA, with a 10:1 labeling ratio.

#### 4.7.4. MST Acquisition

All experimental protein samples were run at 250 nM while varying ds-oligo concentrations (see Methods: Labeling Scheme). Oligos followed a 1:3 (protein:oligo) dilution scheme in PBS-T. After adding 10 µL of labeled protein to each well containing 10µL ds-oligo dilution, the samples were mixed well and incubated for 5–10 min at RT. Hydrophobic capillaries with loaded with ~4 µL protein/oligo mix. Initial runs were conducted at 80% LED and varying MST powers at 20%, 40%, 60%, and 80%. For these samples, 20% MST showed little to no change, 40% MST yielded a good signal, and 60% and 80% did not alter the response with respect to 40% MST. Therefore, the minimum MST at 40% was chosen for analysis. Fluorescence counts were at least 300–600 and always less than 2000. Capscans were inspected and the experiments repeated if the random fluctuation of 20% was exceeded. Sample data were acquired at room temperature (22–26 °C) using the NTControl v2.1.31 software interface.

#### 4.7.5. MST Analysis

After acquiring all of the experimental samples, all of the samples were pooled using MO Affinity Analysis v2.3 and exported as an .ntp file. All samples were loaded into PALMIST 1.4.4 [5] as an .ntp file, and respective experimental replicates were loaded using the .ntp browser for each experimental type. Binding curves were fitted according to a one-to-one binding model. K_D_ fits with the fluorophore fixed to 250 nM (protein concentration) were performed on the average fit unweighted for each measurement zone—which are explained in the main text—using the error surface projection (ESP) method at the 0.683 confidence level. Every individual fit for each experimental combination and measurement zone was exported as a .dat file for graphing in GUSSI [70]. All of the fits for an experimental combination were exported into one Fit Log, and a python script was written to extract all of the values from the Fit Log in order to compile the data for making tables or graph analysis.

### 4.8. Structural Modeling

The GARP2 sequence was aligned to that of the GARP10 and ENG as described by Hosoda and co-workers (2002) [33]. Based on the sequence alignment of GARP2 and GARP10 and the protein structure of GARP10 (1IRZ, first model) as a structural template, we created a homology model for GARP2 using Prime from the Schrödinger Molecular Modeling Platform [90,91]. To obtain protein–DNA complexes for GARP2 and GARP10, we used the protein–DNA complex of ENG (PDB id: 1HDD, chain C) as a template. We superimposed the homology-modeled protein GARP2 onto the protein structure ENG and GARP10 (PDB id: 1IRZ, first model) and onto ENG, respectively, with root mean-square deviation (RMSD)-minimizing superposition using Cα backbone atoms as implemented by the atom bijection method in BALL [92]. In the second step, we created protein–DNA complex structures while taking the DNA from the ENG-protein complex (PDB id: 1HDD, chain A and B) and the protein structures GARP2 (homology model) and GARP10 (PDB id: 1IRZ, first model). We considered amino acid-to-DNA contacts when the distance between protein and DNA atoms (hydrogen atoms not taken into account) fell below a 4.0 Å threshold. The final GARP2 and GARP10 structural models with DNA are available in Supplemental Figures S1–S3.

## Figures and Tables

**Table 1:** Dissociation constants (k_D_) for GARP2–DNA interaction throughout the different MST zones. K_D_ with lower and upper bound values calculated from the binding curves as defined in Figure 5. All parameters were calculated according to a one-to-one binding model using the PALMIST 1.4.4 software. Confidence intervals (C.I.) were calculated via the ESP method (0.683 confidence level). Regions (defined in Figure 4); S.D. (Standard Deviation); Un (unbound). Experiment: GARP2 (His–MBP–GARP2) or His–MBP (MBP) along with the wild-type (wt) or mutated version (mut) of a fragment of the *ARR5* promoter region containing four CRM binding sites.

Type	Fusion	Oligo	Regions	K_D_ [bounds] (nM)	S.D. (nM)
T-jump	GARP2	wt	2/1	93.3 [33.9, 203.5]	40.3
		mut		5422.9 [4271.8, 6928.7]	661.5
	MBP	wt		406.2 [Un, Un]	764.9
		mut		29,278.0 [2846.3, Un]	73167.8
Thermo	GARP2	wt	4_1_/3	455.8 [250.9, 787.3]	131.5
		wt	4_2_/3	525.6 [259.5, 988.2]	178.3
		wt	4_3_/3	519.6 [260.4, 960.9]	172.4
		wt	4_4_/3	462.5 [229.7, 868.7]	163.0
		wt	4_5_/3	510.1 [218.2, 1077.2]	211.7
		wt	4_6_/3	506.0 [192.7, 1173.8]	245.8
		wt	4_7_/3	578.5 [202.0, 1474.6]	316.9
		wt	4_8_/3	679.3 [240.8, 1742.8]	375.6
		wt	4_9_/3	660.3 [200.6, 1912.2]	419.4
		wt	4_10_/3	738.2 [211.1, 2267.5]	504.6
	GARP2	mut	4_1_/3	7621.0 [4510.1, 13,538.4]	2179.6
		mut	4_2_/3	7820.1 [4494.1, 14,274.6]	2247.5
		mut	4_3_/3	8941.7 [4960.4, 17,313.3]	2733.3
		mut	4_4_/3	8617.7 [4314.9, 19,073.8]	3035.9
		mut	4_5_/3	8456.3 [4257.3, 18,824.4]	3061.7
		mut	4_6_/3	8938.1 [3245.4, 33,206.7]	4792.4
		mut	4_7_/3	8121.0 [2446.3, 39,503.1]	5003.2
		mut	4_8_/3	9239.0 [2472.7, 62,858.3]	6153.9
		mut	4_9_/3	6562.0 [1260.5, 50,038.4]	4654.2
		mut	4_10_/3	6602.7 [1179.3, Un]	5150.7
	MBP	wt	4_1_/3	149,761.5 [19,415.2, Un]	1,116,236.4
		wt	4_2_/3	7064.6 [2568.2, 25,579.5]	3852.5
		wt	4_3_/3	4315.4 [1794.4, 10,952.0]	1826.6
		wt	4_4_/3	5387.0 [2993.7, 9992.5]	1583.2
		wt	4_5_/3	5063.2 [2455.4, 11,136.9]	1878.1
		wt	4_6_/3	6648.7 [3395.5, 14,432.7]	2424.9
		wt	4_7_/3	5672.9 [2791.3, 13,055.6]	2399.1
		wt	4_8_/3	6505.9 [3207.5, 15,237.1]	2870.7
		wt	4_9_/3	6462.8 [2914.2, 16,616.1]	3089.9
		wt	4_10_/3	9453.1 [4116.0, 28,162.2]	4808.7
	MBP	mut	4_1_/3	51,212,374.7 [Un, Un]	9,394,498.2
		mut	4_2_/3	58,673,041.9 [Un, Un]	10,990,771.3
		mut	4_3_/3	155,534,044.7 [Un, Un]	14,545,170.7
		mut	4_4_/3	126,324,766.5 [Un, Un]	26,533,955.0
		mut	4_5_/3	166,306,517.3 [Un, Un]	7,086,615.9
		mut	4_6_/3	630,007,997.6 [Un, Un]	3,188,549.4
		mut	4_7_/3	87,588,060.8 [Un, Un]	17,699,022.8
		mut	4_8_/3	581,508,613.5 [Un, Un]	3,533,413.5
		mut	4_9_/3	293,033,378.5 [Un, Un]	5,205,908.5
		mut	4_10_/3	24,422,340.4 [Un, Un]	51,603,646.7
Inv.t-jump	GARP2	wt	6/5	826.5 [471.3, 1412.8]	222.3
		mut		16,158.1 [11,753.7, 23,231.6]	2872.7
	MBP	wt		35,248.3 [721.8, Un]	94,944.9
		mut		37,243,141.3 [Un, Un]	2,581,304.9
Backdiff	GARP2	wt	8/7	166.4 [61.0, 374.4]	74.3
		mut		8516.4 [5019.5, 15,514.5]	2569.0
	MBP	wt		19,483.5 [3759.8, Un]	17,709.6
		mut		604,733,167.6 [Un, Un]	1,193,501.5

## Data Availability

The data and materials presented in this study are available on request from the corresponding authors.

## Supplementary Materials

The following supporting information can be downloaded at: https://www.mdpi.com/article/10.3390/genes14081638/s1: Figure S1: GARP2 structural model; Figure S2: GARP10 DNA complex; Figure S3: GARP2 DNA complex.
